# Analysis of Transcriptional Regulation of the Human miR-17-92 Cluster; Evidence for Involvement of Pim-1

**DOI:** 10.3390/ijms140612273

**Published:** 2013-06-07

**Authors:** Maren Thomas, Kerstin Lange-Grünweller, Dorothee Hartmann, Lara Golde, Julia Schlereth, Dennis Streng, Achim Aigner, Arnold Grünweller, Roland K. Hartmann

**Affiliations:** 1 Institut für Pharmazeutische Chemie, Philipps-Universität Marburg, 35032 Marburg, Germany; E-Mails: thomasm@staff.uni-marburg.de (M.T.); langegru@staff.uni-marburg.de (K.L.-G.); Dorothee_Hartmann@web.de (D.H.); laragolde@gmx.de (L.G.); julia.schlereth@staff.uni-marburg.de (J.S.); dennis.streng@pharmazie.uni-marburg.de (D.S.); 2 Medizinische Fakultät, Rudolf-Boehm-Institut für Pharmakologie und Toxikologie, Klinische Pharmakologie, Universität Leipzig, 04107 Leipzig, Germany; E-Mail: Achim.Aigner@medizin.uni-leipzig.de

**Keywords:** miRNA, miR-17-92 cluster, Pim-1, miRNA promoter, c-Myc, HP1γ, RNAi

## Abstract

The human polycistronic miRNA cluster miR-17-92 is frequently overexpressed in hematopoietic malignancies and cancers. Its transcription is in part controlled by an E2F-regulated host gene promoter. An intronic A/T-rich region directly upstream of the miRNA coding region also contributes to cluster expression. Our deletion analysis of the A/T-rich region revealed a strong dependence on c-Myc binding to the functional E3 site. Yet, constructs lacking the 5′-proximal ~1.3 kb or 3′-distal ~0.1 kb of the 1.5 kb A/T-rich region still retained residual specific promoter activity, suggesting multiple transcription start sites (TSS) in this region. Furthermore, the protooncogenic kinase, Pim-1, its phosphorylation target HP1γ and c-Myc colocalize to the E3 region, as inferred from chromatin immunoprecipitation. Analysis of pri-miR-17-92 expression levels in K562 and HeLa cells revealed that silencing of E2F3, c-Myc or Pim-1 negatively affects cluster expression, with a synergistic effect caused by c-Myc/Pim-1 double knockdown in HeLa cells. Thus, we show, for the first time, that the protooncogene Pim-1 is part of the network that regulates transcription of the human miR-17-92 cluster.

## 1. Introduction

MicroRNAs (miRNAs) are important post-transcriptional riboregulators of gene expression with high relevance to cancer formation and metastasis [1]. In general, miRNAs are derived from RNA polymerase II (RNAPII) primary transcripts (pri-miRNA) that are further processed to ~70 nt precursors (pre-miRNA) and after nuclear export to mature miRNAs by the activity of the two endonucleases, DROSHA/DGCR8 and DICER [2–5]. MiRNAs are incorporated into the miRNA-induced silencing complex (miRISC) and act as repressors of translation by imperfect base-pairing to their target sites in mRNAs [3]. The majority of miRNAs is encoded in intronic regions, either individually or as “polycistronic” miRNA clusters that are cotranscribed [3,6].

Several deregulated miRNAs or miRNA clusters are involved in tumorigenesis, accounting for their designation as tumor-suppressing or as oncogenic miRNAs [7]. Such miRNAs can downregulate targets involved in the regulation of apoptosis or cell cycle progression [1]. One well-characterized polycistronic cluster is the miR-17-92 cluster, also known as OncomiR-1 or C13*orf*25. This cluster encodes six miRNAs belonging to four different seed families: (i) the miR-17 family with miR-17 and miR-20a, (ii) the miR-18 family with miR-18a, (iii) the miR-19 family with miR-19a and miR-19b-1 and (iv) the miR-92 family [8–11]. The human miR-17-92 cluster is encoded in the chromosomal region, 13q31.3, and is amplified in several solid tumors, as well as in some hematopoietic malignancies [8,12]. Because of numerous known targets of its individual miRNAs, the miR-17-92 locus exerts pleiotropic functions during development, proliferation, apoptosis and angiogenesis in different cell systems [13–15]. In mice, deletion of the cluster prevents normal B-cell development as a consequence of premature cell death [14]. In a mouse B-cell lymphoma model, simultaneous overexpression of c-Myc and the miR-17-92 cluster accelerated lymphomagenesis [9]. This oncogenic effect could later be assigned primarily to miR-19a/b, which dampens expression of the tumor suppressor PTEN, thereby repressing apoptosis [13,15].

Analyses of transcriptional regulation of oncogenic miRNAs and miRNA clusters are of great importance for strategies aiming at cancer prevention. Unfortunately, most miRNA promoters have not been characterized or identified yet [16]. In the case of the miR-17-92 cluster, expression is thought to be promoted from a host gene promoter region upstream of exon 1, with transcription starting at a consensus initiator sequence downstream of a non-consensus TATA box [17,18]. Additionally, this core promoter region contains functional E2F transcription factor binding sites. E2F1-3 were shown to activate C13*orf*25 expression from this promoter and chromatin immunoprecipitation assays (ChIP) identified E2F3 to be the main E2F variant associated with the host gene promoter [17,18]. No E2F binding was detected in the region between the host gene promoter and the miR-17-92 cluster [18]. Furthermore, nucleosome mapping combined with chromatin signatures for transcriptionally active promoters [19–21] indicated that transcriptional activity of the miR-17-92 cluster also originates from the intronic A/T-rich region directly upstream of the miRNA coding sequences [16]. This is in line with the finding that cluster expression is activated by c-Myc binding to a conserved E-box element (E3) ~1.5 kb upstream of the miRNA coding sequence [9,10,20]. Indeed, luciferase reporter assays confirmed that both the host gene promoter and the intronic region confer transcriptional activity [16,21].

Here, we subjected the intronic A/T-rich region to deletion analysis using luciferase reporter constructs. Transcription was found to strongly depend on c-Myc binding to the E3 site, but even shorter fragments (<0.3 kb) of sequences directly preceding the miR-17-92 coding sequence still promoted residual, but substantial and specific transcriptional activity. Interestingly, we identified the protooncogene Pim-1 and one of its phosphorylation targets, HP1γ [22], to be associated with the chromatin region containing the E3 site, suggesting that the human C13*orf*25 locus belongs to the set of genes that are regulated by c-Myc and Pim-1 [23,24]. SiRNA-mediated Pim-1 knockdown indeed resulted in reduced pri-miR-17-92 levels, as did a knockdown of c-Myc or E2F3. In Hela cells, a double knockdown of c-Myc/Pim-1 decreased the pri-miR-17-92 levels more than single knockdowns, consistent with a synergism of c-Myc and Pim-1 at the intronic C13*orf*25 promoter.

## 2. Results and Discussion

### 2.1. Results

#### 2.1.1. c-Myc-Dependent Intronic Transcriptional Activity within the Human miR-17-92 Locus

**Figure 1:**
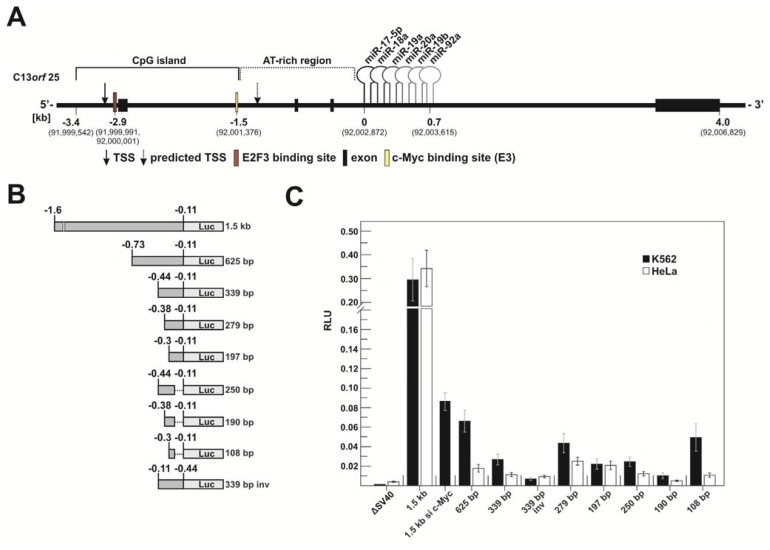
(**A**) Genomic organization of C13*orf*25. The locus consists of four exons and three introns; the six miRNAs of the miR-17-92 cluster are encoded in intron 3. Sequences upstream of the cluster can be subdivided into a G/C-rich CpG island and an A/T-rich downstream part. The host gene promoter thought to be activated by E2F3 is located in the CpG island about 3.4 kb upstream of the miR-17-5p coding sequence. The functional c-Myc site (E3) is located ~1.5 kb upstream of miR-17-5p. Sequence numbering is based on the NCBI reference sequence NG_032702.1 and the GRCh37/hg19 assembly [25]. Note that previous related studies referred to the numbering system of the previous hg18 assembly [16,17,20,21]. The numbering of the hg18 and hg19 assemblies is correlated as follows: nt 92,002,872 (0 kb in Figure 1A) of hg19 is nt 90,800,873 of hg18; (**B**) Schematic representation of the different C13*orf*25 portions fused to the luciferase structural gene. The functional E3 box for c-Myc binding is indicated in the 1.5 kb construct (white vertical line); (**C**) Promoter activities of the different luciferase reporter constructs in K562 and HeLa cells. Obtained luciferase activities were measured as relative light units (RLU) and normalized to the pGL3 control plasmid carrying the SV40 promoter (Promega). A reporter construct lacking the SV40 promoter, as well as a construct harboring the 339 bp fragment of the A/T-rich intronic region in inverted orientation were used as controls. RLU values of the individual constructs were derived from 5 to 16 experiments (+/− S.E.M.).

The 3.4 kb upstream genomic region of the miR-17-92 coding sequence can be subdivided into a G/C-rich and an A/T-rich part. The former is a CpG island (~1.9 kb, 78% GC content; see http://genome.ucsc.edu [25], GRCh37/hg19 assembly) that has its 5′-boundary ~0.4 kb upstream of the TSS of the host gene promoter [20] and its 3′-boundary ~1.4 kb upstream of the miR-17-5p coding sequence, representing the 5′-terminal miRNA of the cluster. The A/T-rich region (~64% A/T content) following the CpG island begins immediately downstream of a functional and highly conserved c-Myc binding site (5′-CATGTG, E-box E3), which is localized ~1.5 kb upstream of the miR-17-5p coding sequence [10] (Figures 1A and S1).

We have analyzed the intronic region of C13*orf*25, including the preceding functional c-Myc box E3 [20] and truncated segments of the A/T-rich region (Figure 1B) for transcriptional activation. For this purpose, luciferase reporter constructs were transfected into K562 (a human myelogenous erythroleukemia cell line from a CML patient) and HeLa cells (an epithelial human cell line from a cervical carcinoma). We selected these two cell lines as a starting point to study transcription of the miR-17-92 cluster in the context of different cellular expression levels (Figure S2).

The ~1.5 kb reporter construct, comprising c-Myc box E3 (Figures 1A and S1) and the A/T-rich region (lacking the 113 bp preceding the mature miR-17-5p coding sequence for reasons of PCR feasibility), showed substantial transcriptional activity, amounting to 30%–35% in both cell lines relative to the pGL3 control plasmid harboring an SV40 promoter (Figure 1C). This is in line with results of a similar study of the mouse miR-17-92 locus [21]. Furthermore, Ozsolak *et al.* [16] predicted an intronic TSS to be localized ~0.2 kb downstream of the E3 site. Indeed, truncating the 1.5 kb fragment to 625 bp, which deletes the E3 site, strongly reduced reporter activity by ~4.5-fold in K562 and by almost 20-fold in HeLa cells compared to the activity of the ~1.5 kb construct (Figure 1C). To substantiate this finding, we tested the ~1.5 kb construct in K562 cells under conditions of a siRNA-mediated knockdown of c-Myc. This reduced reporter expression to a similar extent as the truncation to 625 bp, supporting the notion that c-Myc binding to the E3 site plays a key role in activating transcription from this intronic region (Figure 1C). SiRNA-mediated c-Myc knockdown in HeLa cells also suggests a ~four-fold decrease in transcription originating from the ~1.5 kb reporter construct (data not shown), again consistent with the crucial role of c-Myc binding to the E3 site. As the 625 bp fragment still conferred basal promoter activity, we further shortened this region to ~340 bp, ~280 bp and ~200 bp. Additionally, we included short fragments with their 3′-boundary ~290 bp upstream of the mature miR-17-5p coding sequence (250, 190 and 108 bp in Figure 1B). We also inversed the orientation of the ~340 bp fragment in front of the luciferase gene (Figure 1C, 339 bp inverse (inv)) to include a control fragment with comparable A/T content. This inversed fragment conferred reporter activity 5.3-fold (K562) or 2.4-fold (HeLa) higher than that of the pGL3 control vector lacking the SV40 promoter (ΔSV40, Figure 1C).

All the fragments ≤ 340 bp conferred residual promoter activities, some clearly to a higher extent than the inverted 339 bp fragment in both cell lines (see the 279 and 197 bp fragments, Figure 1C). This indicates that parts of the intronic A/T-rich region promote specific transcriptional activity, the extent partly differing between the two cell lines (Figure 1C). Notably, despite using a variety of web-based promoter prediction tools (see Suppl. Material), no correlation between fragment activity and promoter elements predicted in this region was identified. In K562 cells, the smaller fragments, including the 625 bp fragment, showed an overall trend towards stronger expression relative to HeLa cells.

#### 2.1.2. Pim-1 and HP1γ Are Associated with the Intronic c-Myc Binding Site

**Figure 2:**
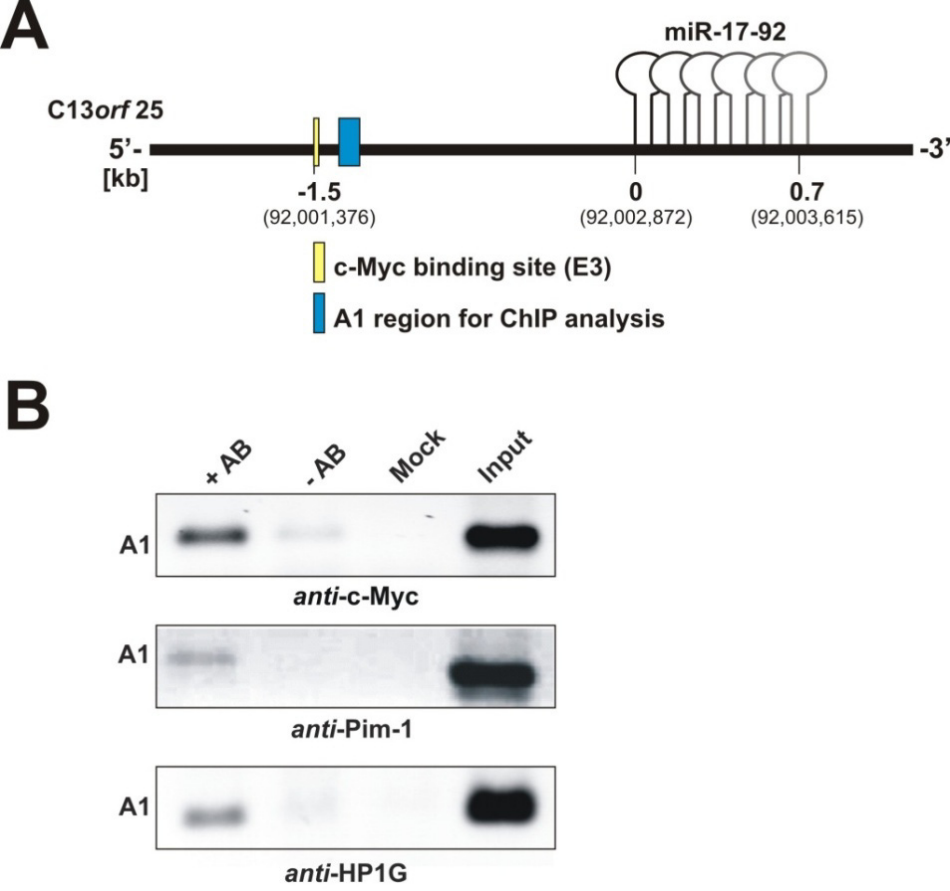
(**A**) Schematic representation of the intronic A/T-rich region preceding the miR-17-92 coding sequence. The region A1 (blue box) defines the genomic sequence 0.1 kb downstream of the functional c-Myc binding site (E3; yellow box) that was amplified in ChIP analyses; (**B**) ChIP analysis of the intronic region A1 in K562 cells, using antibodies specific for c-Myc, Pim-1 and HP1γ. +AB: with antibody; −AB: without antibody; Mock: buffer only without cell lysate; Input: supernatant of the −AB-sample after immunoprecipitation and centrifugation (for details, see Supplementary Materials).

We next asked if other factors beyond c-Myc may be involved in human miR-17-92 cluster expression from the A/T-rich region. Transcriptional regulation by c-Myc is associated with Pim-1-dependent H3S10 phosphorylation in about 20% of all genes regulated by c-Myc [24]. Moreover, Pim-1 and c-Myc act synergistically in severe forms of B-cell lymphomas and Pim-1, as well as the miR-17-92 cluster are overexpressed in K562 cells [26]. We performed ChIP assays to test whether Pim-1 localizes to the internal promoter region of the miR-17-92 cluster. For this analysis*,* we amplified a ~90 bp DNA fragment (segment A1 in Figure 2A) 0.1 kb downstream of the functional c-Myc E3 site. The same DNA segment has been analyzed in a previous study on c-Myc [10]. Our ChIP analysis revealed that not only c-Myc, as expected, but also Pim-1 localizes to this genomic region (Figure 2B, left lanes in upper and middle panels). Indeed, this is consistent with the finding that Pim-1-catalyzed H3S10 phosphorylation is required for c-Myc-dependent transcriptional activation [24]. We further analyzed another known phosphorylation target of Pim-1, the heterochromatin protein-1 gamma (HP1γ) [22], for its association with the E3 region. HP1γ localized to this genomic area, as well (Figure 2B, lower panel). Moreover, we were able to identify an association of HP1γ along the miRNA coding region, which is indicative of active transcription (see Figure S3 and Discussion section).

#### 2.1.3. Transcriptional Activity of the Human miR-17-92 Cluster Depends on c-Myc and Pim-1

**Figure 3:**
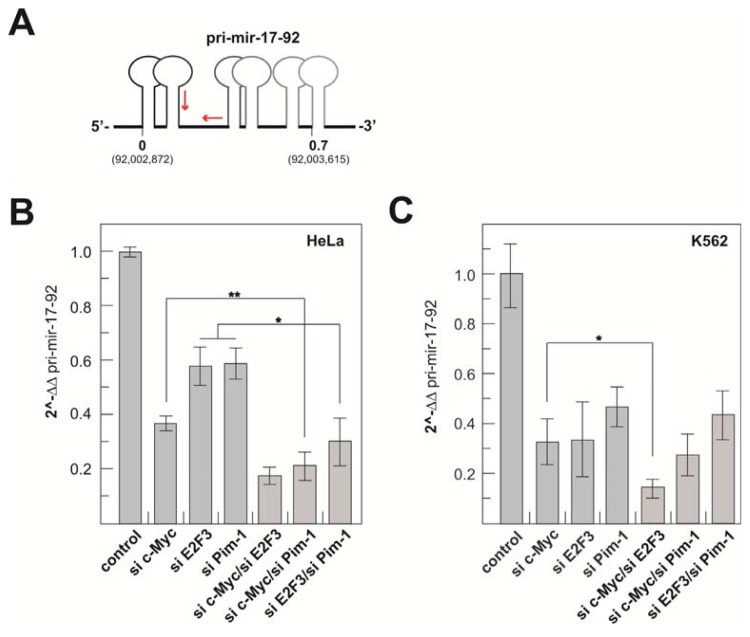
(**A**) Illustration of the primers (red arrows) used for the qRT-PCR quantification of pri-miR-17-92 transcript levels; (**B**,**C**) qRT-PCR-based quantitation of pri-miR-17-92 transcript levels in HeLa (**B**) or K562 cells (**C**) after siRNA-mediated knockdown of c-Myc, E2F3 or Pim-1 or after combined knockdown of c-Myc/E2F3, c-Myc/Pim-1 or E2F3/Pim-1. 2^-ΔΔpri-17-92 values were normalized against 5S rRNA and an internal control siRNA (siVR1), representing mean values from at least three independent experiments (+/− S.E.M.). Statistical analyses were done using the software, R.

To further substantiate the role of Pim-1 in miR-17-92 cluster expression, we quantified the cellular pri-miR-17-92 levels by qRT-PCR (see Figure 3A for primer positions) after siRNA-mediated Pim-1 knockdown relative to a c-Myc knockdown in K562 and HeLa cells. Since combined ChIP and reporter gene assays suggested that the transcription factor E2F3 is a major activator of transcription initiated at the host gene promoter [17,18], we included E2F3 in our knockdown experiments as a possible measure for the contribution of the host gene promoter to miR-17-92 expression. We also quantified the levels of c-Myc, E2F3 and Pim-1 mRNAs after knockdown by qRT-PCR to evaluate knockdown efficiencies (Supplementary Table S1). For Pim-1, we have shown good correlation between mRNA and protein levels [26], suggesting that reduced mRNA levels will also entail decreased protein levels. A corresponding parallel analysis of protein levels was inconclusive, owing to a non-interpretable pattern obtained with the used E2F3 antibody [18]. In the study presented here, only experiments with a knockdown efficiency >50% were included (Supplementary Table S1). Single knockdowns of either c-Myc or E2F3 decreased the pri-miR-17-92 levels in HeLa cells to ~35 and 60%, respectively, relative to the control siRNA (Figure 3B). Notably, a 40% reduction of pri-miR-17-92 levels was also observed upon Pim-1 knockdown. Similar results were obtained in K562 cells, with decreases in pri-miR-17-92 levels to ~30%, 30% and 45%, respectively (Figure 3C). However, double knockdowns had additive suppression effects on pri-miR-17-92 levels in the case of c-Myc/E2F3 (HeLa and K562), c-Myc/Pim-1 (Hela) and E2F3/Pim-1 (HeLa). To shed more light on the role of Pim-1, we further analyzed luciferase activity of the 1.5 kb construct harboring the functional c-Myc E3 site in K562 and HeLa cells upon Pim-1 knockdown. We did not observe a substantial decrease in reporter expression after Pim-1 knockdown in K562 cells (data not shown), but a three-fold reduction (Supplementary Figure S4) in HeLa cells (see Discussion).

### 2.2. Discussion

The transcription of the oncogenic miR-17-92 cluster is thought to originate from two different TSSs: one is localized in close proximity to the host gene promoter element [17] (Supplementary Figure S1), and the other TSS was predicted to map to the region ~200 bp downstream of the functional c-Myc site E3 (Figures 1A and S1). The latter prediction was based on nucleosome mapping and chromatin signatures for active promoters. The derived algorithm identified 175 human promoters proximal to miRNA coding sequences and was reported to correctly predict transcription initiation regions to a resolution of 150 bp with high sensitivity and specificity. The majority of predictions were also consistent with known “expressed sequence tag” (EST) TSSs or cDNA 5′-ends [16].

Beyond previous studies [16,20,21], we investigated the intronic A/T-rich region preceding the human miR-17-92 cluster in more detail and compared it to siRNA-mediated knockdown of c-Myc. Similar effects were obtained, substantiating the notion that c-Myc and the c-Myc E3 site play a crucial role in activating transcription from the intronic promoter region. However, the 625 bp and even some of the further truncated fragments (~280 and ~200 bp) of the A/T-rich region conferred residual specific promoter activity in both cell types (Figure 1), indicating that parts of the A/T-rich region, downstream of the c-Myc E3 site, contribute to cluster expression. This E3 box-independent transcriptional activity was more pronounced for K562 relative to HeLa cells, which correlates with the particularly high cluster expression in K562 cells (Supplementary Figure S2). As a possible explanation, transcriptional activity of the ~1.5 kb fragment may be dominated by the recruitment of c-Myc to the E3 site region, while differential activity mediated by the smaller fragments in K562 *vs.* HeLa cells may report that their residual transcriptional activation is mechanistically different from that of the ~1.5 kb fragment. This could mean that regulatory factors of the transcription machinery are differentially expressed in the two cell lines.

ChIP assays revealed that not only c-Myc, but also the protooncogene Pim-1 and its phosphorylation target, HP1γ, associate with the chromatin region harboring the c-Myc E3 site (Figure 2B). Importantly, Pim-1-catalyzed phosphorylation of H3S10 at c-Myc target genes is necessary to regulate key genes required for c-Myc-dependent oncogenic transformation [27].

In mammals, three paralogs of HP1 (α, β and γ) regulate heterochromatin formation, gene silencing or gene activation [28,29]. HP1α and β proteins are mainly recruited to heterochromatin regions harboring H3K9me2,3 modifications, whereas HP1γ is found in association with euchromatin [30] and active genes [29]. Furthermore, HP1c, the *Drosophila* homolog of HP1γ, associates with transcriptionally active chromatin containing H3K4me3 and H3K36me3 histone marks [28]. HP1γ can further be recruited to inducible promoters, where it replaces HP1β, thereby inducing a switch from the repressive to the active transcriptional state. This replacement with HP1γ requires H3 phospho-acetylation [31]. In this context, a transient phosphorylation of H3S10 (via Aurora B kinase) was shown to be necessary for the dissociation of HP1 proteins from chromatin during the M phase of the cell cycle [32]. In the induced state, HP1γ can also be localized within coding regions of protein genes, together with elongating RNA polymerase II [31].

Our data, showing that HP1γ colocalizes with Pim-1 and c-Myc (Figure 2), is in line with the aforementioned activating role of HP1γ during transcription. We extended our ChIP assays to the miRNA coding region of C13*orf*25 to analyze HP1γ association with this part of the cluster. Indeed, ChIP analysis along the miRNA coding sequence identified HP1γ at all four analyzed subregions (A2–A5, Supplementary Figure S3). To our knowledge, this is the first indication that HP1γ is involved in activating the transcription of miRNAs.

The association of Pim-1 with the intronic chromosomal region near the c-Myc E3 site led us to the assumption that Pim-1 plays an important role in the transcriptional activation of the miR-17-92 cluster. This was tested by RNAi also, including E2F3 as an assumed indicator of host gene promoter activity. The strong negative effects of individual knockdowns of c-Myc, Pim-1 and E2F3 on pri-miR-17-92 levels indicate that all three proteins are important for cluster expression by affecting transcription from the host gene promoter (E2F3) or the intronic promoter region (c-Myc, Pim-1). This raises the question about the mechanistic role of Pim-1 in cluster expression from the intronic promoter. In contrast to HeLa cells Supplementary (Figure S4), a Pim-1 knockdown in K562 cells failed to significantly decrease reporter activity from the ~1.5 kb fragment. Among other possibilities, Pim-1 may be recruited to the functional c-Myc E3 site in the context of the cellular chromatin structure in K562 cells, but not in the context of the plasmid-encoded reporter gene. Alternatively, Pim-1 recruitment to the E3 site occurs, as shown by the ChIP assays, but is not a crucial prerequisite for transcriptional activation in K562 cells. On the other hand, the three-fold decrease in ~1.5 kb reporter activity observed in HeLa cells upon Pim-1 depletion adds evidence in support of a crucial role for Pim-1 in miR-17-92 cluster expression, but simultaneously points to cell type-specific differences. For future investigations, other cell lines will be tested, particularly ones that express c-Myc, but not Pim-1. Clearly, decreases in pri-miR-17-92 levels upon c-Myc, E2F3 and/or Pim-1 knockdown (Figure 3) may include indirect effects, e.g., originating from inhibition of cell proliferation (Pim-1), changes in the kinetics of pri-miR-17-92 processing, global changes in transcriptional networks (E2F3, c-Myc) or mutual transactivation (E2F3 and c-Myc) [33–35]. Further complication may arise from the fact that miR-17-5p and miR-20a of the cluster are negative regulators of E2F1-3 mRNAs [10,18].

As c-Myc, HP1γ and histone H3 are known phosphorylation targets of Pim-1, future studies may address the influence of Pim-1 on the phosphorylation status of these proteins at the E3 site, utilizing antibodies that are highly specific for the phosphorylated *versus* unphosphorylated state.

The siRNA-mediated c-Myc knockdown, decreasing c-Myc mRNA levels on average by 65% (HeLa) or 81% (K562; see Supplementary Table S1), resulted in a 60%–70% reduction in pri-miR-17-92 levels in HeLa and K562 cells (Figure 3). This effect may report a rough estimate of the proportion of cluster transcripts normally initiated in the intronic promoter region in these two cell lines, for the following reasons: the C13*orf*25 region contains four c-Myc binding sites (boxes E1-4) and two additional ones with lower c-Myc occupancy (relative to E1) upstream of the host gene promoter [20]. Box E1, immediately downstream of host gene promoter’s TSS, was shown by deletion analysis to be inhibitory, which correlates with c-Myc forming heterodimers with MXI or MNT at this site to repress transcription [20]. Thus, host gene promoter activity may even somewhat increase under conditions of a c-Myc knockdown, although such an effect could, in turn, be neutralized by reduced c-Myc-mediated transactivation of E2F [35]. ChIP-Seq data for K562 and HeLa-S3 cells revealed the by far highest c-Myc occupancy at site E3 (little at E2 and E4), where c-Myc forms heterodimers with MAX to activate transcription [20]. A straightforward interpretation of the additive effect of a c-Myc/E2F3 double knockdown in Hela and K562 cells is that this combination negatively affected transcription from the host gene and intronic promoter regions.

A major finding of our study is the recruitment of Pim-1 to the intronic c-Myc E3 site (Figure 2) and the strong negative effect of a Pim-1 knockdown on cluster expression (Figure 3B,C). Interestingly, Pim-1 knockdown efficiencies are comparable in K562 (73%) and HeLa (71%) cells, whereas the effect of the knockdown on cluster expression is stronger in K562 cells (55% reduction compared to 40% in HeLa cells) with the higher Pim-1 expression level. This might be due to cell type-dependent indirect effects of Pim-1 on the regulation of the miR-17-92 cluster. Moreover, double knockdown experiments in HeLa cells revealed a synergistic effect relative to individual c-Myc and Pim-1 knockdowns (Figure 3B), which was not seen for K562 cells. The siRNA-mediated reduction of c-Myc and Pim-1 mRNAs were on average 86% and 77% in HeLa and 86% and 52%, respectively, in K562 cells (Supplementary Table S1). The somewhat weaker suppression of Pim-1 in the c-Myc/Pim-1 double knockdown context (cf. with single knockdowns, Table S1) in K562 *versus* HeLa cells may have contributed to the absence of a clear additive effect upon c-Myc/Pim-1 double knockdown in K562 cells.

## 3. Experimental Section

### 3.1. Oligonucleotides

Small interfering RNAs (siRNAs) were purchased from Dharmacon (Boulder, CO, USA):

VR1 siRNA [36] was used as an unrelated negative control, with the following sequences of sense and antisense strand.

VR1 siRNA sense 5′-GCG CAU CUU CUA CUU CAA CdTdT and antisense 5′-GUU GAA GUA GAA GAU GCG CdTdT.

The sequences of all other siRNAs used in this study are:

Pim-1 siRNA sense 5′-GAU AUG GUG UGU GGA GAU AdTdT and antisense 5′-UAU CUC CAC ACA CCA UAU CdTdT; Pim-1 siRNA 2 sense→5′-GGA ACA ACA UUU ACA ACU CdTdT and antisense 5′-GAG UUG UAA AUG UUG UUC CdTdT; c-Myc siRNA sense 5′-CAG GAA CUA UGA CCU CGA CUA dTdT and antisense 5′-UAG UCG AGG UCA UAG UUC CUG dTdT; E2F3 siRNA sense 5′-ACA GCA AUC UUC CUU AAU AdTdT and antisense 5′-UAU UAA GGA AGA UUG CUG UdTdT.

### 3.2. Antibodies

Antibodies against c-Myc (sc-40) and Pim-1 (sc-13513), as well as the secondary antibody (sc-2005: goat anti-mouse IgG HRP conjugated) were purchased from Santa Cruz Biotechnology (Heidelberg, Germany) except for the Phospho HP1γ (Ser83) antibody (2600S), which was obtained from Cell Signaling Technology (Danvers, MA, USA).

### 3.3. Plasmid Construction and Seed Mutagenesis

For the construction of promoter-luciferase fusions, the SV40 promoter of plasmid “pGL3 control” (Promega, Mannheim, Germany) was removed via digestion with *Bgl*II and *Hind*III (Thermo Fisher Scientific, Schwerte, Germany) and replaced with fragments derived from the intronic A/T-rich region of C13*orf*25 (reference nucleotide sequence NG_032702.1). Promoter fragments were amplified from genomic DNA of the human cell line K562 using primers specified in the Supplementary Material. PCR products were purified using the Wizard^®^ SV Gel and PCR Clean-Up System (Promega, Mannheim, Germany) and digested with *Bgl*II and *Hind*III for insertion into pGL3. All constructs were cloned in *E. coli* DH5α cells and verified by DNA sequencing. The pGL3 vector lacking the SV40 promoter, as well as the pGL3 construct carrying the C13*orf*25-derived 339 bp fragment in inverse orientation (pGL3 339 bp inv), were used as negative controls.

### 3.4. Transfection Procedures and Luciferase Reporter Assays

Assays are described in detail in the Supplementary Materials.

### 3.5. Chromatin Immunoprecipitation

Chromatin immunoprecipitation (ChIP) was performed according to a protocol from the Or Gozani lab at Stanford University (http://www.stanford.edu/group/gozani) [37], with some modifications. For details, see the Supplementary Material.

## 4. Conclusions

We report here that miR-17-92 cluster expression from the intronic A/T-rich promoter region, although critically depending on c-Myc binding, includes some specific contribution of sequences within ~0.7 kb upstream of the mature miR-17-5p coding sequence. Our reporter expression data suggest multiple TSSs within this A/T-rich region, although the transcription initiation region predicted by Ozsolak *et al.* [16], ~0.2 kb downstream of the c-Myc E3 box, may well be the major one. E3 site-independent transcription initiation within ~0.7 kb upstream of the mature miR-17-5p coding sequence was more pronounced in K562 *versus* HeLa cells (Figure 1), indicating cell type-specific differences in cluster expression from the intronic promoter region. By RNAi and ChIP, we establish that Pim-1 acts in concert with c-Myc at the E3 site to activate transcription from the intronic promoter region.

## Supplementary Figures

Figure S1 Relevant sequence region of C13*orf*25, including the CpG island harboring the host gene promoter, the A/T-rich region, and the miR-17-92 cluster; sequence and position of the last exon 4 of C13*orf*25 is indicated at the end. Shown sequences are based on the NCBI reference sequence NG_032702.1 and the GRCh37/hg19 assembly [25]. The boundaries of the CpG island, important previously identified regulatory elements, mature miRNA coding sequences and relevant primer sequences are highlighted in the sequence and annotated at the margins.

Figure S2 Quantitative RT-PCR of the pri-mir-17-92 transcription levels in the human cell lines K562, HeLa and HUH7 (hepato cellular carcinoma cells). 2^-ΔΔpri-mir-17-92 values are normalized against 5S rRNA and obtained from at least 3 independent experiments (+/−S.E.M.). The amount of pri-mir-17-92 transcript in K562 cells was set to 1.

Figure S3 (**A**) Schematic representation of the intronic A/T-rich region preceding the miR-17-92 coding sequence. The region A1 defines the genomic sequence 0.1 kb downstream of the functional c-Myc binding site (E3 box) that was amplified in ChIP analyses. A2 covers a segment immediately upstream of the miRNA-coding region; A3–A5 are located along the coding sequence of the human miR-17-92 cluster. The length (bp) of each amplicon is indicated at the top; (**B**) ChIP analysis of the regions A1 to A5 in K562 cells, using an antibody specific for HP1γ or RNA polymerase II (only A2 analyzed). +AB, with antibody; −AB, without antibody; Mock, buffer only without cell lysate; Input, supernatant of the “−AB” sample after immunoprecipitation and centrifugation (for details, see Supplementary Material); (**C**) ChIP analysis of the A2 region in HeLa cells using the antibody specific for HP1γ.

Figure S4 Effect of an siRNA-mediated Pim-1 knockdown on promoter activity of the ~1.5 kb reporter construct in HeLa cells. RLU values were derived from 5 independent triplicate experiments (+/−S.D.). RLU values for the control (left bar, transfected with the reporter plasmid but in the absence of a siRNA) were set to 100%. Lipofectamine transfection of HeLa cells was done as described under Supplementary methods, with the following alterations: 2 × 10^5^ cells were used, and 40 pmol (0.6 μg) siRNA plus 0.5 μg of the reporter plasmid were combined in 50 μL Opti-MEM^®^ I medium and mixed with 1.5 μL Lipofectamine™ 2000 in 50 μL Opti-MEM^®^ I medium. The resulting mixture (~100 μL) was incubated for 20 min at room temperature to allow complex formation before addition to the cells. For the control (left bar), the siRNA was omitted.

## Supplementary Table

**Table S1:** Quantification of the pri-miR-17-92 levels and the c-Myc, E2F3 and Pim-1 mRNA levels after siRNA-dependent knockdown by qRT-PCR. Expression levels were calculated from the crossing points by the 2^-ΔΔ*C*_T_ method [38] using β-Actin mRNA or 5S rRNA as internal controls. To determine the c-Myc, E2F3 and Pim-1 knockdown efficiencies, expression levels were normalized to the levels obtained by transfection of K562- or HeLa cells with an unrelated siRNA directed against the vanilloid receptor (siVR1).

K562	c-Myc KD		E2F3 KD		Pim-1 KD	

	2^-Δ ΔC _T_ pri-mir-17-92	KD efficiency	2^-Δ ΔC _T_ pri-mir-17-92	KD efficiency	2^-Δ ΔC _T_ pri-mir-17-92	KD efficiency
	0.13	77	0.21	95	1.23	72
	0.02	87	0.16	98	0.16	69
	0.56	59	0.60	19	1.39	55
	0.75	74			0.76	59
	0.33	75			0.22	49
	0.14	99			2.38	78
	0.20	97			1.84	87
	0.55	80			0.80	88
					0.27	85
					0.40	89
					0.52	67
					0.69	65
					0.37	87

**Mean**	**0.33**	**81**	**0.34**	**89**	**0.47**	**73**
SEM	0.09	5.0	0.15	8.0	0.08	5.0

K562	c-Myc/E2F3 KD		c-Myc/Pim-1 KD		Pim-1/E2F3 KD	

	2^-Δ ΔC _T_ pri-mir-17-92	KD efficiency	2^-Δ ΔC _T_ pri-mir-17-92	KD efficiency	2^-Δ ΔC _T_ pri-mir-17-92	KD efficiency
	0.22	82/75	0.45	72/55	0.69	88/86
	0.23	86/72	0.19	90/54	0.56	68/73
	0.15	81/48	0.19	95/48	0.19	95/99
	0.08	99/99			0.52	87/81
	0.04	99/99			0.23	91/93

**Mean**	**0.15**	**89/79**	**0.28**	**86/52**	**0.44**	**86/86**
SEM	0.04	4.0/9.6	0.08	7.0/2.2	0.09	4.7/4.5

HeLa	c-Myc KD		E2F3 KD		Pim-1 KD	

	2^-Δ ΔC _T_ pri-mir-17-92	KD efficiency	2^-Δ ΔC _T_ pri-mir-17-92	KD efficiency	2^-Δ ΔC _T_ pri-mir-17-92	KD efficiency
	0.37	70	0.72	51	0.50	47
	0.29	82	0.66	78	0.34	72
	0.32	61	0.40	76	0.71	63
	0.41	99	0.53	73	0.58	80
	0.48	55	0.64	73	0.54	87
	0.37	64			0.78	72
					0.70	76

**Mean**	**0.37**	**65**	**0.59**	**70**	**0.59**	**71**
SEM	0.03	6.7	0.07	6.3	0.06	4.9

HeLa	c-Myc/E2F3 KD		c-Myc/Pim-1 KD		Pim-1/E2F3 KD	

	2^-Δ ΔC _T_ pri-mir-17-92	KD efficiency	2^-Δ ΔC _T_ pri-mir-17-92	KD efficiency	2^-Δ ΔC _T_ pri-mir-17-92	KD efficiency
	0.24	79/64	0.24	79/84	0.49	81/96
	0.13	91/81	0.25	77/61	0.33	91/85
	0.11	93/86	0.15	86/58	0.10	97/94
	0.12	90/91	0.12	95/91	0.26	92/94
	0.23	87/74	0.41	84/73		
	0.26	83/66	0.10	97/95		

**Mean**	**0.18**	**87/77**	**0.21**	**86/77**	**0.30**	**90/92**
SEM	0.03	2.2/4.4	0.053	3.3/6.0	0.08	3.3/2.5

## Appendix

### Cell Culture

Cells (K562 and HeLa) were cultivated under standard conditions in a humidified atmosphere (37 °C, 5% CO_2_) supplemented with RPMI 1640 (K562) or IMDM (HeLa) containing 10% FCS (PAA, Cölbe, Germany).

### Chromatin Immunoprecipitation (ChIP)

2 × 10^7^ K562 cells in 13 mL RPMI medium were crosslinked with 1% formaldehyde for 10 min at 37 °C. Reactions were stopped by adjusting to 0.125 M glycine, and cells were collected by centrifugation at 400*g* for 5 min at room temperature. For cell lysis, cells were resuspended in 750 μL RIPA-buffer (10 mM Tris-HCl pH 7.4, 150 mM NaCl, 1% deoxycholate, 1% NP40, 0.1% SDS, 0.2 mM PMSF) supplemented with the complete Mini Protease Inhibitor Mix from Roche (Mannheim, Germany) according to the manufacturer’s instructions. Lysed cells were sonicated in a Branson Sonifier 250 (duty cycle 50%, output control 2, for 3.5 min, on ice water) (Heinemann, Schwäbisch Gmünd, Germany) and centrifuged at 16,000*g* for 10 min at 4 °C. The supernatant was pre-cleared with 10 μL of blocked *Staphylococcus aureus* cells (Pansorbin^®^ Cells, Calbiochem/Merck, Darmstadt, Germany) for 15 min at 4 °C on a rotor wheel. After a second centrifugation step (16,000*g*, 5 min, room temperature), the supernatant was split into two samples (each ~350 μL, representing + and − specific antibody (AB)), which were adjusted to buffer D (16.7 mM Tris-HCl pH 8.1, 167 mM NaCl, 1.2 mM EDTA, 1.1% Triton-X 100, 0.01% SDS) and a total volume of 500 μL. 1 μg (1 to 5 μL) of the respective antibody was added to “+AB” samples, whereas the same volume buffer D was added to “−AB” samples. At this point, also the “Mock” control was prepared, consisting of 500 μL buffer D. “+AB”, “−AB” and “Mock” samples were then incubated for at least 3 h at 4 °C. The following antibodies were used: monoclonal anti-c-Myc (sc-40) and anti-Pim-1 (sc-13513) (Santa Cruz Biotechnology (Heidelberg, Germany), anti-Phospho HP1γ (Ser83) polyclonal antibody (2600S) (Cell Signaling Technology, Danvers, MA, USA). In the case of mouse monoclonal antibodies (c-Myc and Pim-1), samples were additionally incubated for 1 h with a second monoclonal goat anti-mouse IgG antibody (sc-2005, Santa Cruz Biotechnology, Heidelberg, Germany). Immunoprecipitation was initiated by adding 10 μL of Pansorbin^®^ cells (see above) to the “+AB”, “−AB” and “Mock” samples, followed by incubation for 15 min at room temperature. Samples were centrifuged (16,000× *g*, 3 min, room temperature); the supernatant of the “−AB” sample was saved, later serving as the input control. Pellets were washed twice with dialysis buffer (50 mM Tris-HCl pH 8.0, 2 mM EDTA) and four times with IP-wash buffer (100 mM Tris-HCl pH 9.0, 500 mM LiCl, 1% NP40, 1% deoxycholate). Antibody-bound material was eluted from Pansorbin^®^ cells by adding 150 μL elution buffer (50 mM NaHCO_3_, 1% SDS), vortexing for 3 s, and centrifugation (16,000 *g*, 3 min, room temperature). The supernatant was collected and the procedure was repeated. Reverse crosslinking and RNA digestion was performed in 280 μL buffer [0.3 M NaCl, and 1 μL RNase A (10 mg/mL)] for 5 h at 67 °C. Chromatin was precipitated with ethanol, followed by a Proteinase K digest (Thermo Fisher Scientific, Bremen, Germany). DNA was purified by phenol/chloroform extraction and ethanol precipitation in the presence of 0.3 M NaOAc, pH 5.2. PCR amplification using Taq DNA polymerase and the co-immunoprecipitated DNA as template was done under the following conditions: 2 min at 95 °C in the absence of enzyme, followed by 35 amplification cycles of 45 s at 95 °C/45 s at 60 °C/45 s at 72 °C using the following primers:

A1 forward 5′-AAA GGC AGG CTC GTC GTT G
A1 reverse 5′-CGG GAT AAA GAG TTG TTT CTC CAA
A2 forward 5′-ACA TGG ACT AAA TTG CCT TTA AAT G
A2 reverse 5′-AAT CTT CAG TTT TAC AAG GTG ATG
A3 forward 5′-ACT GCA GTG AAG GCA CTT GT
A3 reverse 5′-TGC CAG AAG GAG CAC TTA GG
A4 forward 5′-CCA ATA ATT CAA GCC AAG CAA
A4 reverse 5′-AAA TAG CAG GCC ACC ATC AG
A5 forward 5′-GCC CAA TCA AAC TGT CCT GT
A5 reverse 5′-CGG GAC AAG TGC AAT ACC AT

### Transfection of Reporter Constructs

For transfection of the suspension cell line K562, cells were washed in medium without serum, followed by electroporation in a BioRad Gene Pulser XCell (München, Germany) with a single pulse in a 4 mm cuvette, using 5 μg of the respective pGL3 derivative plasmid per million cells. 48 h after transfection, cells were washed in PBS, lysed and prepared for luciferase reporter assay measurements.

### Transfection of siRNAs

Transfection of the suspension cell line K562 was performed by electroporation. After a washing step in medium without serum, 10^6^ cells were mixed with 1 μg of siRNA (VR1 siRNA, Pim-1 siRNA, c-Myc siRNA or E2F3 siRNA). For the double knockdown experiments, 1 μg of each siRNA, respectively, was used. Cells were electroporated at 330 V with a single pulse in a BioRad Gene Pulser XCell (München, Germany) using a 4 mm cuvette. After electroporation, K562 cells were resuspended in medium containing 10% FCS and cultivated in a humidified atmosphere at 37 °C for 24 h. Cells were washed with PBS and prepared for total RNA extraction.

Transfection of HeLa cells was done using the transfection agent Lipofectamine™ 2000 (Life Technologies Invitrogen, Darmstadt, Germany). One day before transfection, 8 × 10^4^ cells were seeded into 24-well plates and cultivated under standard conditions. SiRNA complexes (VR1 siRNA, Pim-1 siRNA, c-Myc siRNA or E2F3 siRNA) were prepared according to the manufacturer’s protocol. To perform single knockdown experiments, 40 pmol of the respective siRNA were used. In case of the double knockdown experiments, 20 pmol of each siRNA were mixed in Opti-MEM^®^ 1 (Life Technologies Invitrogen, Darmstadt, Germany). 4 to 6 h after transfection, the medium was replaced with IMDM containing 10% FCS. Cells were cultivated 48 h under standard conditions until preparation for total RNA extraction.

### RNA Preparation and Quantitative Real-Time PCR

For total RNA isolation, transfected cells (K562 or HeLa) were lysed (vortexing or mixing by pipetting up and down) in 750 μL lysis solution (0.8 M guanidinium-thiocyanate, 0.4 M ammonium-thiocyanate, 0.1 M sodium acetate pH 5.0, 5% glycerin, 38% phenol pH 4.5–5.0 (Roth^®^-Aqua-Phenol, Roth, Karlsruhe, Germany), 1 pellet 8-hydroxychinolin). Then, 200 μL of chloroform was added and phases were separated by centrifugation. The aqueous phase was mixed with 2 volumes of isopropanol, followed by incubation for 15 min at room temperature and centrifugation. The air-dried RNA pellet was dissolved in RNase-free water and incubated for 30 min at 37 °C with 1 U DNase I per μg RNA in 100 μL 1× DNase I buffer (DNase I, Thermo Fisher Scientific, Schwerte, Germany) according to the manufacturer’s instructions. Then, another identical aliquot of DNase I was added, followed by incubation at 37 °C for another 30 min. Samples were extracted with an equal volume of Roth®-Aqua-Phenol (see above), followed by extraction of the aqueous phase with chloroform and isopropanol precipitation as above. RNA pellets were finally washed with 75% ethanol, air-dried and redissolved in 10 μL RNase-free water. 0.5 to 1 μg of total RNA were reverse-transcribed with RevertAid H Minus RT Polymerase (Thermo Fisher Scientific) according to the manufacturer’s protocol. For determination of KD efficiencies a random hexamer primer was used to generate cDNA samples. In case of calculating pri-mir-17-92 levels the gene-specific reverse primer specified below was used for cDNA synthesis. Quantitative RT-PCR was performed in duplicate in a BioRad iQ™5 (BioRad, München, Germany) with the Absolute qPCR SYBR Green Capillary Mix (Thermo Scientific AbGene, Hamburg, Germany); cDNAs were diluted 1:5 or 1:10 and 4 μL of the reaction mixture used for determining RNA transcription levels. Quantitative PCR assays for miRNA detection were conducted as follows: Thermo-Start™ DNA polymerase was activated for 15 min at 95 °C followed by 55 amplification cycles of 10 s at 95 °C/20 s at 60 °C/12 s at 72 °C. Subsequently, melting curves of the PCR products were generated: samples were cooled from 95 to 65 °C (20 °C per s), kept at 65 °C for 20 s, followed by heating steps of 1 °C per cycle up to 95 °C and kept for 10 s at 95 °C.

Quantitative RT-PCR assays for mRNA detection were changed as follows: Thermo-Start™ DNA polymerase was activated for 15 min at 95 °C followed by 55 amplification cycles with a denaturation step for 10 s, primer annealing for 10 s at 55 °C and amplification at 72 °C for 10 s. Subsequently, a melting curve was generated for the PCR products; samples cooled from 95 to 65 °C (20 °C per s), kept at 65 °C for 20 s, followed by heating steps of 1 °C per cycle up to 95 °C and kept for 10 s at 95 °C.

The mRNA and pri-miR-17-92 levels were calculated from the crossing points by the 2^-ΔΔ*C*_T_ method [38] using β-Actin mRNA or 5S rRNA as internal controls. Knockdown efficiency was quantitated by qRT-PCR and only those experiments showing more than 65% reduction in protein levels were used for quantification of pri-miR-17-92 levels. All primers for qPCR measurements were purchased from Metabion (Martinsried, Germany) and designed using the software tool Universal ProbeLibrary (Roche Applied Biosystems, Mannheim, Germany). A list of all primer sequences according to the human sequences is shown underneath:

Pri-miR-17-92: forward primer 5′-CAT CTA CTG CCC TAA GTG CTC CTT and reverse primer 5′-GCT TGG CTT GAA TTA TTG GAT GA;
5S rRNA: 5′-TCT CGT CTG ATC TCG GAA GC and 5′-AGC CTA CAG CAC CCG GTA TT;
c-Myc mRNA: forward primer 5′-CCT TGC AGC TGC TTA GAC and reverse primer 5′-GAG TCG TAG TCG AGG TCA T;
E2F3 mRNA: forward primer 5′-GAG ACT GAA ACA CAC AGT CC and reverse primer 5′-CCT GAG TTG GTT GAA GCC;
Pim-1 mRNA: forward primer 5′-ATC AGG GGC CAG GTT TTC T and reverse primer 5′-GGG CCA AGC ACC ATC TAA T;
Actin mRNA: forward primer 5′-CCA ACC GCG AGA AGA TGA and reverse primer 5′-CCA GAG GCG TAC AGG GAT AG.

### Luciferase Reporter Assays

Luciferase reporter assays were performed using the Promega Luciferase Assay System (Promega, Mannheim, Germany). After aspirating the medium, cells were washed with PBS and lysed in 100 μL 1.2× reporter lysis buffer. In a 96-well plate, 10 μL of the respective cell lysate were mixed with 10 μL of luciferase substrate. Chemiluminescence was measured immediately in a Safire^2™^ micro-plate reader (Tecan, Crailshaim, Germany).

### Plasmid Construction and Seed Mutagenesis

All primers, which were used for plasmid construction and seed mutagenesis, are listed below (restriction sites in italics):

pGL3 1.5 kb: 5′-ATA T*AG ATC T*TG CCG CCG GGA AAC GGG TT and reverse primer R1 5′-ATA T*AA GCT T*CC ATA CAA ATT CAG CAT AAT CCC TAA TGG;
pGL3 625 bp: 5′-ATA TAG ATC TCT TTA GAC AAT GTA CCT TTT CTG and reverse primer R1 (see above);
pGL3 339 bp: 5′-ATA T*AG ATC T*GT GGA AGC CAG AAG AGG AGG A and reverse primer R1 (see above);
pGL3 279 bp: 5′-ATA TAG ATC TGG TAC ACA TGG ACT AAA TTG CC and reverse primer R1 (see above);
pGL3 197 bp: 5′-ATA T*AG ATC T*CT CTA TGT GTC AAT CCA TTT GGG AG and reverse primer R1 (see above);
pGL3 250 bp: 5′-ATA T*AG ATC T*GT GGA AGC CAG AAG AGG AGG A and reverse primer R3 5′-ATA T*AA GCT T*GC CTT AAG AAT TCT TTA CAG AAG GC;
pGL3 190 bp: 5′-ATA T*AG ATC T*GG TAC ACA TGG ACT AAA TTG CC and reverse primer R3 (see above);
pGL3 108 bp: 5′-ATA T*AG ATC T*CT CTA TGT GTC AAT CCA TTT GGG AG and reverse primer R3 (see above);
pGL3 339 inv: 5′-ATA T*AA GCT T*GT GGA AGC CAG AAG AGG AGG A and 5′-ATA T*AG ATC T*CC ATA CAA ATT CAG CAT AAT CCC TAA TGG.

### Bioinformatical Promoter Analysis

For promoter analyses of the intronic A/T-rich region, a sequence of 1490 bp (human), beginning at the functional c-Myc site (E3) and ending at the 5′-end of the first mature miRNA sequence, miR-17-5p (for details, see Supplementary Figure S1), was analyzed with several web-based promoter prediction tools. The following tools were used to predict promoter elements or putative TSSs:

*Neural Network promoter prediction:* (http://www.fruitfly.org/seq_tools/promoter.html) [39];
*McPromoter006:* (http://tools.genome.duke.edu/generegulation/McPromoter/) [40];
*Promoter 2.0 Prediction Server:* (http://www.cbs.dtu.dk/services/Promoter/) [41];
*PromPredict:* (http://nucleix.mbu.iisc.ernet.in/prompredict/prompredict.html) [42].

Putative TSSs were predicted and calculated using the software available at http://rulai.cshl.org/tools/genefinder/CPROMOTER/human.htm[43].

All promoter prediction tools predicted several different promoter elements in the 1.5 kb region upstream of miR-17-5p and only the web-based tool Promoter 2.0 failed. The calculated promoter predictions of the indicated tools did not match in any sequence region, giving the assumption that the A/T-rich 1.5 kb intronic region in front of the miR-17-92 coding sequence has only weak promoter activity itself. Due to the fact that this intronic region has an overall high A/T-content, nearly all software programs were able to detect putative promoter regions.

### Statistical Analysis

Statistical analyses were done using the software R [44]. *p*-Values were calculated with the Welch Two Sample Test.
