# Supplementary figures and images for: Analysis of Transcriptional Regulation of the Human miR-17-92 Cluster; Evidence for Involvement of Pim-1
Source: Int J Mol Sci. 2013 Jun 7;14(6):12273–96. doi: 10.3390/ijms140612273 (PMC3709785; doi:10.3390/ijms140612273)

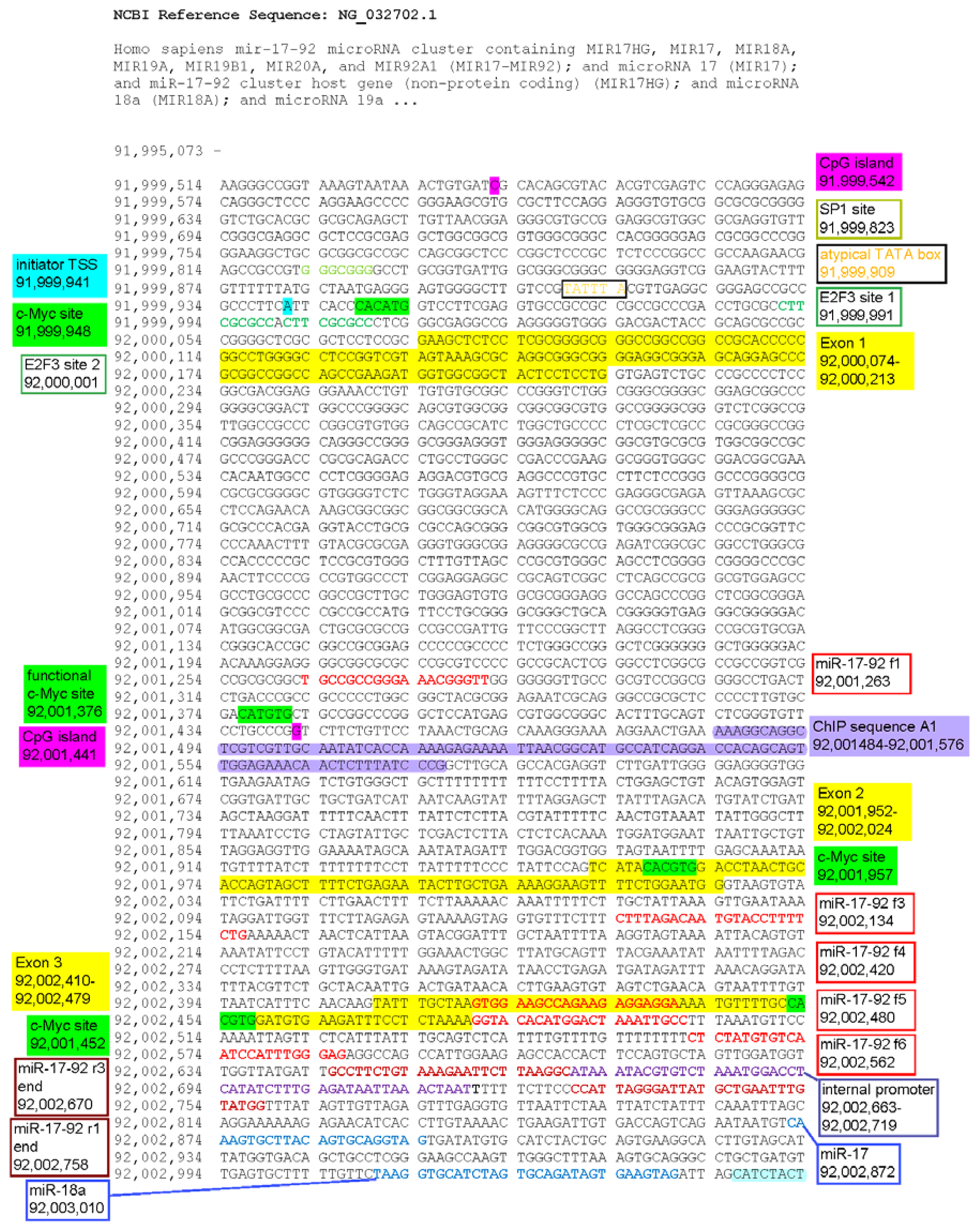

Supplement: Figure S1 — Relevant sequence region of C13orf25, including the CpG island harboring the host gene promoter, the A/T-rich region, and the miR-17-92 cluster; sequence and position of the last exon 4 of C13orf25 is indicated at the end. Shown sequences are based on the NCBI reference sequence NG_032702.1 and the GRCh37/hg19 assembly [25]. The boundaries of the CpG island, important previously identified regulatory elements, mature miRNA coding sequences and relevant primer sequences are highlighted in the sequence and annotated at the margins. [file ijms-14-12273s1a.tif]

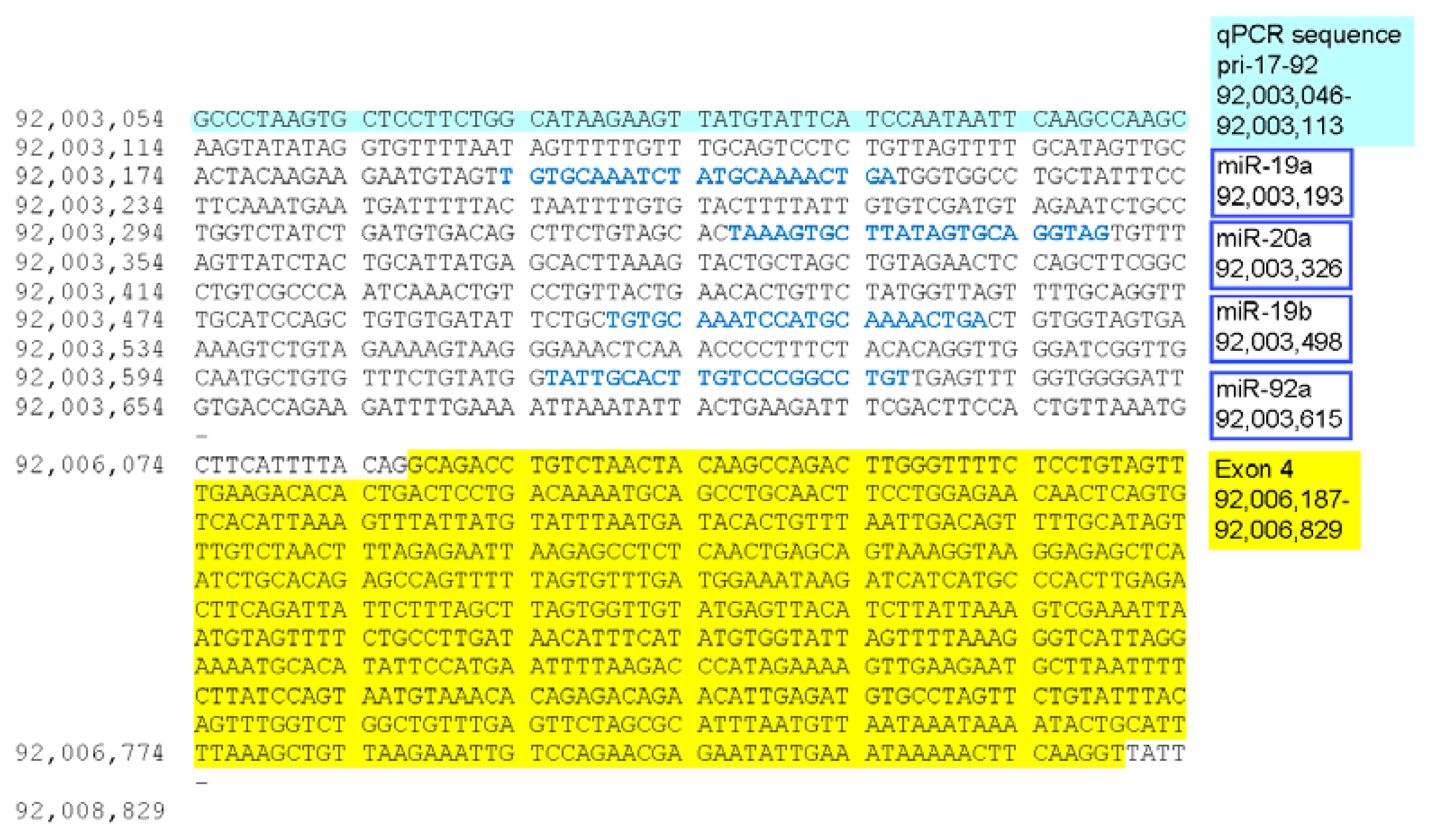

Supplement: Figure S1 — Relevant sequence region of C13orf25, including the CpG island harboring the host gene promoter, the A/T-rich region, and the miR-17-92 cluster; sequence and position of the last exon 4 of C13orf25 is indicated at the end. Shown sequences are based on the NCBI reference sequence NG_032702.1 and the GRCh37/hg19 assembly [25]. The boundaries of the CpG island, important previously identified regulatory elements, mature miRNA coding sequences and relevant primer sequences are highlighted in the sequence and annotated at the margins. [file ijms-14-12273s1b.tif]

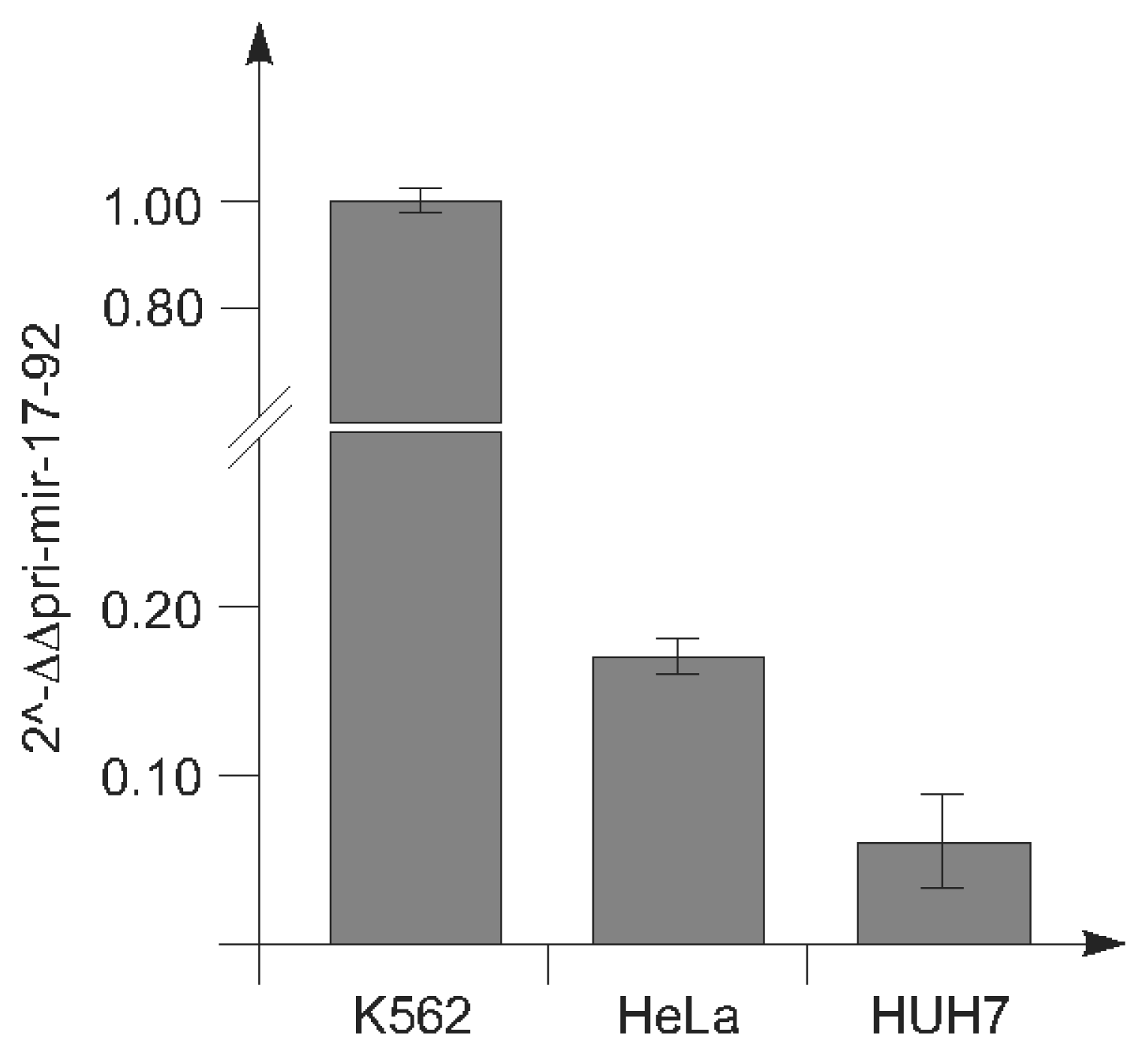

Supplement: Figure S2 — Quantitative RT-PCR of the pri-mir-17-92 transcription levels in the human cell lines K562, HeLa and HUH7 (hepato cellular carcinoma cells). 2^-ΔΔpri-mir-17-92 values are normalized against 5S rRNA and obtained from at least 3 independent experiments (+/−S.E.M.). The amount of pri-mir-17-92 transcript in K562 cells was set to 1. [file ijms-14-12273s2.tif]

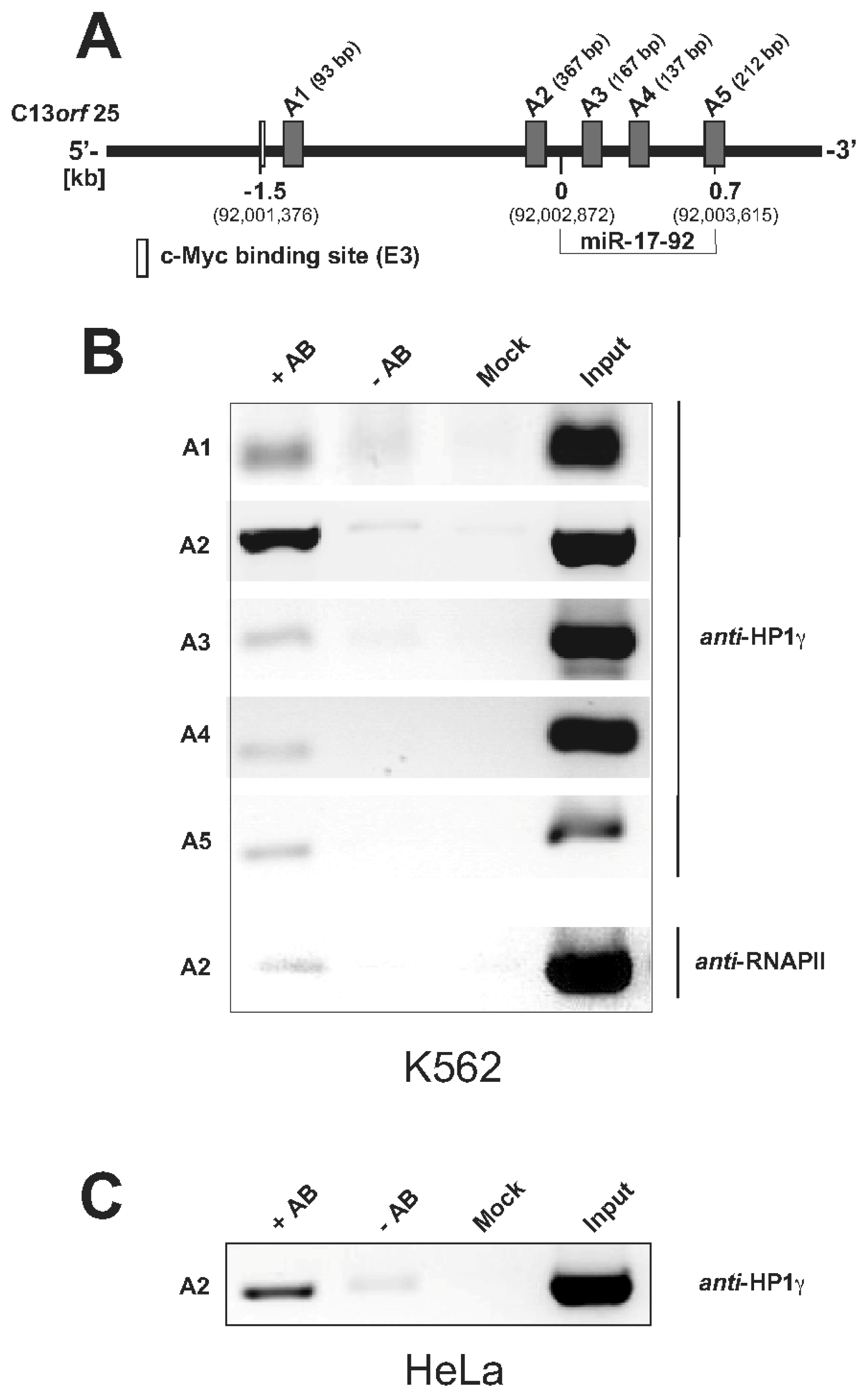

Supplement: Figure S3 — (A) Schematic representation of the intronic A/T-rich region preceding the miR-17-92 coding sequence. The region A1 defines the genomic sequence 0.1 kb downstream of the functional c-Myc binding site (E3 box) that was amplified in ChIP analyses. A2 covers a segment immediately upstream of the miRNA-coding region; A3–A5 are located along the coding sequence of the human miR-17-92 cluster. The length (bp) of each amplicon is indicated at the top; (B) ChIP analysis of the regions A1 to A5 in K562 cells, using an antibody specific for HP1γ or RNA polymerase II (only A2 analyzed). +AB, with antibody; −AB, without antibody; Mock, buffer only without cell lysate; Input, supernatant of the “−AB” sample after immunoprecipitation and centrifugation (for details, see Supplementary Material); (C) ChIP analysis of the A2 region in HeLa cells using the antibody specific for HP1γ. [file ijms-14-12273s3.tif]

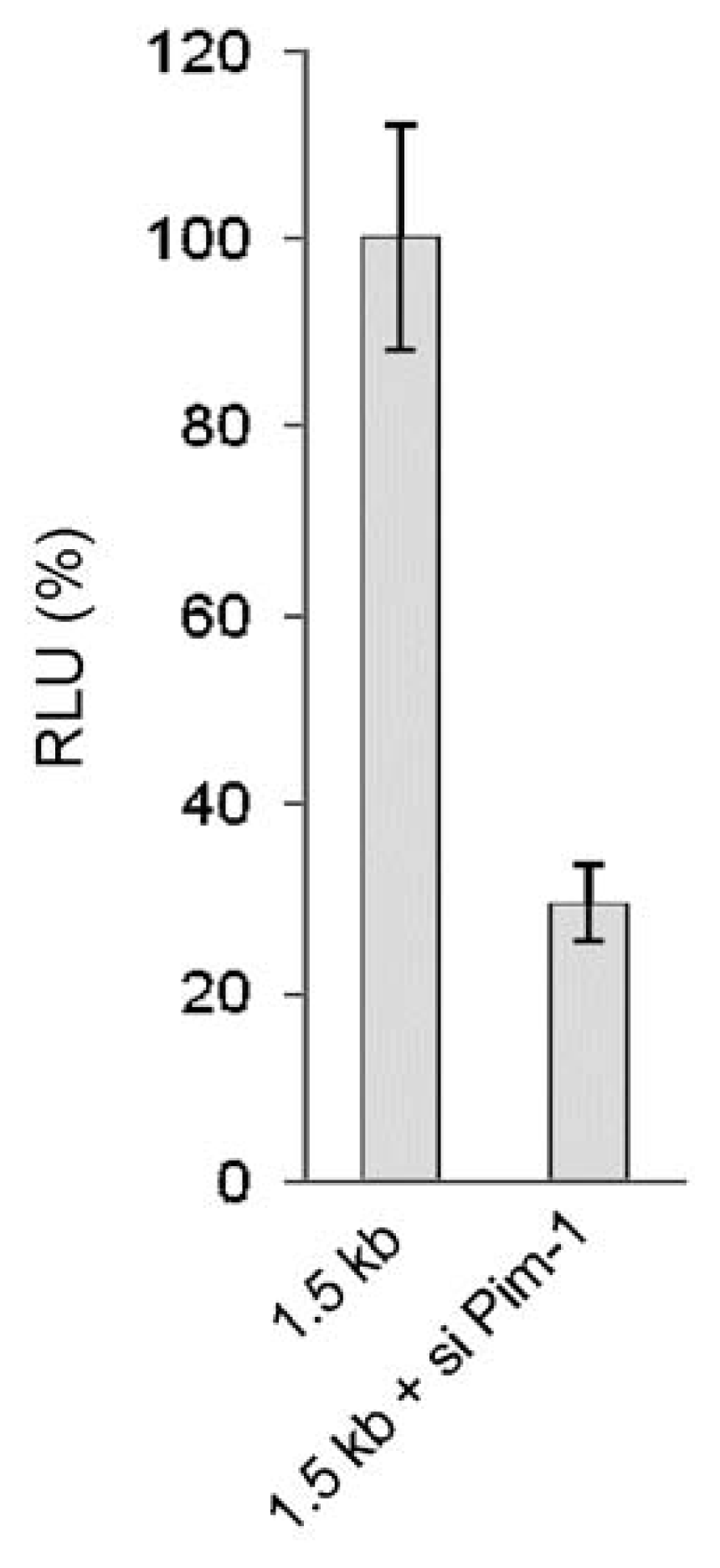

Supplement: Figure S4 — Effect of an siRNA-mediated Pim-1 knockdown on promoter activity of the ~1.5 kb reporter construct in HeLa cells. RLU values were derived from 5 independent triplicate experiments (+/−S.D.). RLU values for the control (left bar, transfected with the reporter plasmid but in the absence of a siRNA) were set to 100%. Lipofectamine transfection of HeLa cells was done as described under Supplementary methods, with the following alterations: 2 × 10^5^ cells were used, and 40 pmol (0.6 μg) siRNA plus 0.5 μg of the reporter plasmid were combined in 50 μL Opti-MEM® I medium and mixed with 1.5 μL Lipofectamine™ 2000 in 50 μL Opti-MEM® I medium. The resulting mixture (~100 μL) was incubated for 20 min at room temperature to allow complex formation before addition to the cells. For the control (left bar), the siRNA was omitted. [file ijms-14-12273s4.tif]
